# Simple Phenotypic Sweeps Hide Complex Genetic Changes in Populations

**DOI:** 10.1093/gbe/evv004

**Published:** 2015-01-13

**Authors:** Ram P. Maharjan, Bin Liu, Lu Feng, Thomas Ferenci, Lei Wang

**Affiliations:** ^1^School of Molecular Bioscience, University of Sydney, New South Wales, Australia; ^2^TEDA Institute of Biological Sciences and Biotechnology, Nankai University, Tianjin, People's Republic of China; ^3^Key Laboratory of Molecular Microbiology and Technology, Ministry of Education, Tianjin, People’s Republic of China; ^4^State Key Laboratory of Medicinal Chemical Biology, Nankai University, Tianjin, People’s Republic of China

**Keywords:** experimental evolution, mutational sweeps, metagenomics, *E. coli* genome, periodic selection

## Abstract

Changes in allele frequencies and the fixation of beneficial mutations are central to evolution. The precise relationship between mutational and phenotypic sweeps is poorly described however, especially when multiple alleles are involved. Here, we investigate these relationships in a bacterial population over 60 days in a glucose-limited chemostat in a large population. High coverage metagenomic analysis revealed a disconnection between smooth phenotypic sweeps and the complexity of genetic changes in the population. Phenotypic adaptation was due to convergent evolution and involved soft sweeps by 7–26 highly represented alleles of several genes in different combinations. Allele combinations spread from undetectably low baselines, indicating that minor subpopulations provide the basis of most innovations. A hard sweep was also observed, involving a single combination of *rpoS*, *mglD*, *malE*, *sdhC,* and *malT* mutations sweeping to greater than 95% of the population. Other mutant genes persisted but at lower abundance, including *hfq*, consistent with its demonstrated frequency-dependent fitness under glucose limitation. Other persistent, newly identified low-frequency mutations were in the *aceF, galF, ribD* and *asm* genes, in noncoding regulatory regions, three large indels and a tandem duplication; these were less affected by fluctuations involving more dominant mutations indicating separate evolutionary paths. Our results indicate a dynamic subpopulation structure with a minimum of 42 detectable mutations maintained over 60 days. We also conclude that the massive population-level mutation supply in combination with clonal interference leads to the soft sweeps observed, but not to the exclusion of an occasional hard sweep.

## Introduction

Once upon a time, the concept of mutational sweeps was relatively simple. It was assumed that individual beneficial mutations sweep to fixation in bacterial populations before another mutation arises in the fitter mutant and in turn increases in frequency (Atwood et al. 1951a). This periodic selection notion tied in nicely with ecological principles that clones excluded others from a single niche (Armstrong and McGehee 1980), so the supposed mutational successions reflected this search for the fittest in one environment. This mode of adaptation also depends on the selective sweep originating from a single new mutation, this scenario being called a “hard” sweep (Hermisson and Pennings 2005). A further consequence of such hard sweeps is that ancestral variation that is unlinked to the selected allele will be eliminated. An example of this early view, relevant to the experimental system we use, is the statement that “evolution in continuous cultures usually consists of a succession of dominant strains of increasing growth rate” (Kubitschek 1970; Dykhuizen and Hartl 1983).

Views on mutational adaptation in large populations have shifted in recent years and suggest that adaptation is more likely to produce “soft” selective sweeps, where multiple adaptive alleles sweep through the population at the same time, because the alleles either were already present as standing genetic variation or arose independently by recurrent de novo mutations (Pennings and Hermisson 2006; Messer and Petrov 2013). A source of standing genetic variation comes from the concept of clonal interference (Gerrish and Lenski 1998; de Visser and Rozen 2006), which reduces the elimination of competing mutations in large populations. A combination of clonal interference and soft sweeps has been used to explain events in experiments with replicate yeast populations (Lang et al. 2013) and several other independent studies are inconsistent with successions and hard sweeps.

For example, a pure culture of a bacterium in a constant, stirred, single-resource continuous culture environment rapidly diversified (Maharjan et al. 2006), contrary to historical assumptions about successions and niche exclusion in a highly controlled environment. Diverse lineages were the reproducible outcome of *Escherichia coli* growth in glucose- or phosphate-limited continuous cultures (Kinnersley et al. 2009; Wang et al. 2010; Maharjan et al. 2012) as are glucose-limited yeast populations (Gresham et al. 2008). The divergence in single-resource chemostats is apparent in 60–100 generations and populations eventually form consortia after longer-term maintenance in a glucose-limited chemostat (Rosenzweig and Adams 1994). This result is inconsistent with hard sweeps being the sole recurring mode of adaptation in large bacterial populations. Rapid clonal divergence is evident also in nature, in clonal infections by *E. coli* (Levert et al. 2010) as well as in the diversity of phenotypes generated by the same mechanisms as are operating in chemostats (Maharjan, Nilsson, et al. 2013; Phan and Ferenci 2013).

Another set of observations inconsistent with hard sweeps in a chemostat population came from genome sequencing of individual highly represented clones (Kinnersley et al. 2009; Maharjan et al. 2010, 2012; Gaffe et al. 2011). By identifying beneficial mutations in different isolates, it was clear that multiple mutational lineages arose in chemostats directly from ancestor, so multiple, concurrent changes in allele frequency are more likely to be the norm. Equally, adaptation did not start from a single new mutation as is expected from a hard sweep; these findings are also more consistent with soft sweeps where more than a single copy of the allele contributes to an adaptive substitution (Hermisson and Pennings 2005; Pennings and Hermisson 2006). Once distinct mutational lineages are present in a population, complex trade-offs, epistasis, and frequency-dependent interactions provide the basis of coexistence of bacteria in the same environment (Maharjan and Ferenci 2013; Maharjan, McKenzie, et al. 2013).

Evidence supporting soft sweeps has recently emerged from metagenomic studies using experimental populations of various microbes (Herron and Doebeli 2013; Kvitek and Sherlock 2013; Lang et al. 2013). These are also consistent with independent lineages emerging with yeast and *E. coli*. From the above accumulating evidence, it would seem that a core feature of evolution, sweeps by fitter types within populations, is more complex than simply sweeps to complete fixation (Barrick and Lenski 2013). Here, we study the detailed kinetics of allele frequency changes in an evolving *E. coli* chemostat population to allow a detailed understanding of events such as the extent of fixation of mutations by soft and hard sweeps and the time-scale of concurrent sweeps in fixed environments. The nature of classical, so-called periodic selection events (Atwood et al. 1951b) causing fluctuations in the proportion of neutral alleles in populations (Koch 1974; Berg 1995; Notley-McRobb and Ferenci 2000) needs to be reconciled with what is really happening in large populations. We here attempt to define such events using metagenomic analysis.

A further aim here is a comprehensive description of the mutational changes under glucose limitation because previous mutation analysis was biased by sequencing only high-abundance members of chemostat populations. In a chemostat system we use, a large population of 10^10^ bacteria is under constant strong selection for the improved utilization of the limiting nutrient (Ferenci 2008a). Three mutations have been previously identified in replicate populations and increased in representation to greater than 50% of the population by about 100 generations (Notley-McRobb et al. 2003; Ferenci 2008b). Mutations in these *rpoS*, *mglD*/O, and *malT* genes were of considerable competitive benefit under glucose limitation (Notley-McRobb and Ferenci 1999a, 1999b). More recently, *hfq* mutations have also been found in most but not all populations (Maharjan et al. 2010); these, in contrast to the three above, are present in less than 50% of population members and show negative frequency dependence in fitness assays (Maharjan, McKenzie, et al. 2013). Epistasis prevents the occurrence of *hfq* and *rpoS* mutations in the same background so the *rpoS* and *hfq* lineages persist in parallel (Maharjan and Ferenci 2013).

To avoid biases against low-abundance types and to overcome this limitation, we here used a high-coverage metagenomic analysis of a glucose-limited chemostat population over 60 days, which allows us to reveal the sweeps and persisting low-level subpopulations as well as to identify novel genes mutated in chemostats. In this study, we follow the temporal distribution of all mutations represented at and above 1% frequency at multiple time-points and analyze the genetic basis of major changes in population composition and the alleles responsible.

Advances in genomics now allow very high coverage analysis of sequences in a population. Although metagenomics is mostly applied to environmental samples rather than pure cultures, it has the potential to reveal all of the changes in populations starting from a pure clone (Barrick and Lenski 2009; Herron and Doebeli 2013; Kvitek and Sherlock 2013; Lang et al. 2013). Here, we apply metagenomics to 11 samples over 60 days of adaptation to obtain a high-resolution analysis of events in a bacterial culture evolving in a homogeneous environment. The very high sequence coverage in our analysis has now permitted the identification of many new mutations and the observation that many mutations persist over time but in less than 5% abundance in the population. These low-proportion mutations are not directly impacted by the major sweeps so provide new evidence for coevolving subpopulations in a homogeneous environment. Subpopulations also, as shown here, provide the recurring source of innovation in a large bacterial population.

## Materials and Methods

### Bacterial Strain and Growth Conditions

For the experimental evolution, the *E. coli* K-12 strain BW2952 (Ferenci et al. 2009), an MC4100 derivative, was propagated at the growth (dilution) rate of 0.1 h^−^^1^ in a glucose-limited chemostat exactly as described in Notley-McRobb et al. (2003).

### Phenotypic Changes in the Population

One phenotypic change in the evolving chemostat population was detected by using the iodine staining method as described previously in Notley and Ferenci (1995). Staining darkness is indicative of the level of expression of the general stress response in *E. coli* (Maharjan, Nilsson, et al. 2013). Briefly, 100-µl aliquots of 10^5^-fold-diluted chemostat cultures were plated on L-broth agar plates in duplicate and incubated for overnight at 37 °C. Plates containing 250–300 colonies were then transferred into a fridge (4 °C) and kept for 24 h before flooding with iodine solution. Colonies were then scored depending on their staining. Colonies with an *rpoS* mutation (no stress response) stay yellow in color, whereas colonies with high levels of *rpoS* stain dark (Maharjan, Nilsson, et al. 2013).

A second phenotype commonly changed in chemostats is the level of *mal* expression (Notley-McRobb and Ferenci 1999b). To measure this phenotype, we assayed the *malG–lacZ* reporter fusion β-galactosidase activity incorporated into the ancestral BW2952 using Miller Units as described in Ferenci et al. (2009). Bacteria from glycerol cultures stored from each time-point were transferred into 5 ml Luria broth (LB) and grown overnight to stationary phase. β-Galactosidase activities of cultures were then measured as described previously in Notley and Ferenci (1995).

### Mutator Phenotype Assay

For qualitative mutator phenotype assay, a single colony of isolates to be tested was inoculated in 5 ml LB. After growing to stationary phase at 37 °C, 100 µl of culture directly spread onto LB plates containing rifampicin at 100 µl/ml. For total viable count, 100 µl of appropriately diluted cultures in LB were plated in LB-agar plates. The plates were then incubated overnight at 37 °C. The number of rifampicin-resistant colonies in each plate was counted. Isolates producing resistant colonies more than 10-fold in number relative to the ancestor were considered as mutator.

### DNA Preparation for Population Level Whole-Genome Sequencing

For chromosomal DNA samples, 40 ml cultures from different time points were collected through the waste outlet of the evolving chemostat culture and harvested by centrifugation at 3,000 × g for 5 min at 4 °C. Cell-pellets were resuspended in 750 µl of TE buffer (10 mM Tris and 1 mM ethylenediaminetetraacetic acid, pH = 8.0) and treated with 50 µl of lysozyme (20 mg/ml) for 15 min at 37 °C. An amount of 15 µl proteinase K (20 mg/ml) and 25 µl sodium dodecyl sulfate (20% wt/vol) was added into lysozyme-treated cell suspensions and incubated for further 1–3 h or until suspensions became clear. The DNA was then extracted using phenol–chloroform method as previously described (Ferenci et al. 2009). All chemicals used for DNA preparation were purchased from Sigma-Aldrich, Australia.

### Genomics Details

The genome resequencing workflow started with sample preparation, which generated sequencer-ready DNA fragments of paired-end libraries. Solexa Genome Analyzer IIx (Illumina, Little Chesterford, Essex, UK) was used to sequence each sample aiming for 1,000-fold coverage.

The 101-bp Solexa paired-end FASTQ reads generated, after being trimmed by SolexaQA (v.1.12) (Cox et al. 2010), contiguous segments longer than 25 bp in which the probability value of each base greater than 0.05. Then, polymerase chain reaction (PCR) duplicates were filtered out by removing pairs with the same first 20 bases in both reads. All nonredundant read pairs were aligned to the *E. coli* BW2952 genome using BWA (Li and Durbin 2009) with the default parameter, which generated the sequence alignment map (SAM) file.

Freebayes (Garrison and Marth 2012) with –pooled-discrete enabled and SAMtools with default parameters (Li et al. 2009) were used to detect the SNPs (single-nucleotide polymorphisms), small indels (insertions and deletions), and small complex events (composite insertion and substitution events) for each position and the total read depth at the locus. Small genetic variants, including SNPs, single base pair or small indels (S-indels) and small complex events in each sequenced sample were called at positions covered by sequences of both the forward strand and the reverse stand, and by at least three reads supporting the same mutant allele. Allelic frequencies of each mutation were then estimated from the number of reads and the total number of coverage.

The method for detecting SNPs was verified by analyzing the resequencing data (100-fold coverage) for *E. coli* BW2952 and MG1655, which was generated by the same sequencing method as that for other samples in this study. No alternate allele due to sequencing error was found anywhere in these genomes.

Large structural variant events, such as large deletions, medium-sized insertions, inversions, and tandem duplications, were detected from the alignment using Pindel (v0.2.4) (Ye et al. 2009), with a pattern growth algorithm to identify the breakpoints of these variants from the paired-end short reads.

## Results

### Genomic Changes in an Evolving Chemostat Population

In order to understand how phenotypic and genotypic sweeps emerge in evolving asexual populations, we performed genomic analysis of a whole population initially propagated from a single ancestral *E. coli*. This strain was the same as that used in our previous lab evolution studies (Notley-McRobb and Ferenci 1999a, 1999b; Ferenci et al. 2009). The glucose-limited population of approximately 1.6 × 10^10^ was studied over 60 days and samples cultures stored at −80 °C. Whole-population DNA samples were however extracted from bacteria taken directly from the chemostat during the 60 days so that anomalies caused by freezing and reviving cultures did not change the genome composition.

The 11 samples obtained over 60 days were subjected to whole-population genomic sequencing as described in the Materials and Methods section. The average coverage of libraries in sequencing was 800-fold. After the informatics filtering for PCR artifacts and errors in sequencing, we tested the possible occurrence of false positives by subjecting genomes from two *E. coli* pure cultures (of the ancestor BW2952 and the reference strain MG1655) to the same sequencing and filtering methods. No mutations relative to published corrected genome sequences were observed. This provided confidence that the mutations identified in the population samples were not sequencing artifacts.

**Fig. 1.—:**
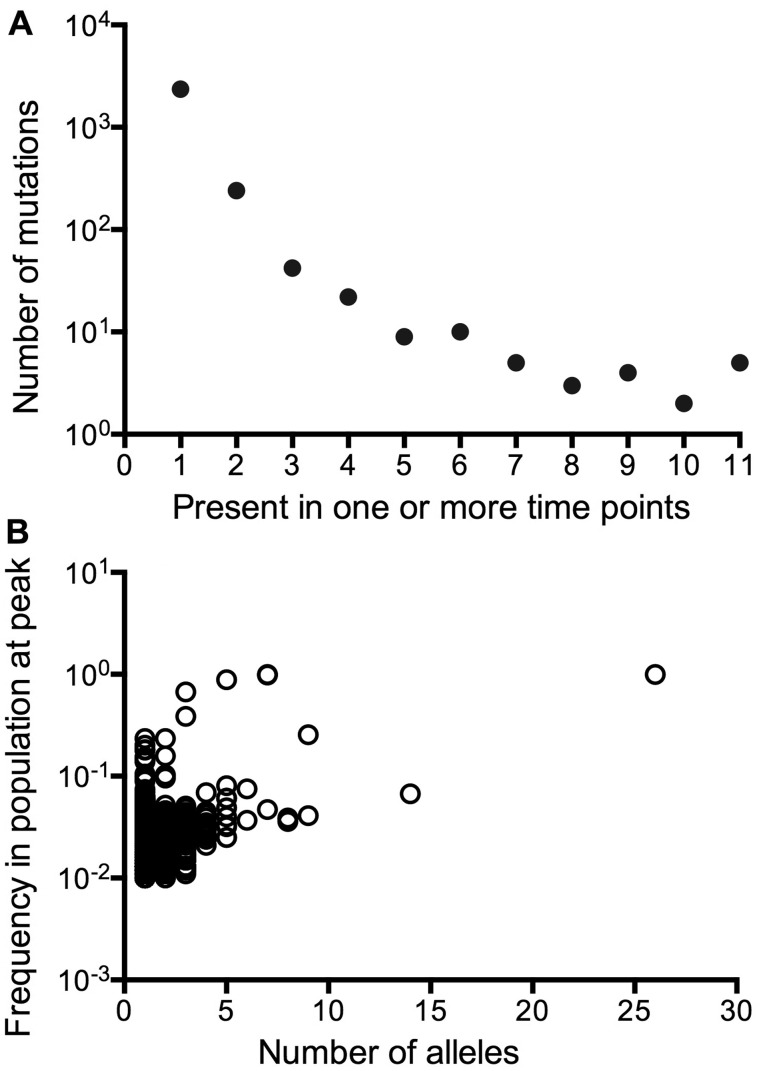
Distribution of mutations detected by whole-genome sequencing of an evolving population. (*A*) The number of occurrences of mutations within 11 samples. The histogram was based on a total of 2,729 mutations (1,182 SNPs, 74 small indels, 4 large indels, and 1 duplication mutation) across 11 samples collected over 60 days of continuous culture. (*B*) The relationship between the number of alleles found in mutated genes and their frequency at peak in the population. The histogram was based on 2,439 mutations in 1,435 genes with one or more mutations.

In total, we detected 2,729 changes from the ancestor (2,664 SNPs, 45 single base-pair indels, 4 large indels, and 1 duplication) in the 11 samples. All mutations, as well as their allelic frequencies at each time point, are listed in supplementary table S1, Supplementary Material online. The large majority of mutations (2,366 or 87%) were present only once (fig. 1*A*), of which 1,974 mutations were present in 1–2% of the population. This represents more than 10^8^ bacteria of 1.6 × 10^10^ cells in the evolving population but did not become fixed in the population. Only 343 (13%) of the total 2,729 mutations were detected in multiple time samples, of which 32 (11%) mutations peaked to constitute over 10% of the population. The most abundant in frequency in the population were the recurrent beneficial mutations in particular genes with multiple alleles (fig. 1*B*). In contrast, singleton mutations were generally of lower abundance but large in number.

Of the 2,664 SNPs, 2,415 are located in coding regions of which 1,696 (64%) and 719 (27%) are nonsynonymous and synonymous SNPs, respectively. The remaining 249 (9%) SNPs are located in noncoding regions. All genes with nonsynonymous mutations that repeatedly occurred at more than three time points are listed in table 1. These are the recurrent mutations often with multiple alleles in figure 1*B*. The recurrence of the mutations in table 1, even at low abundance, suggests that these are likely to be real population components. So below, we discuss the occurrence of mutations established enough to be repeatedly found in greater than three time-points at or above 1% abundance.

**Table 1:** Nonsynonymous Changes Present at Three or More Time-Points in Nonrepeat Regions of the Genome^a^

Gene Name	Function	Occurrence in How Many Samples	Number of Alleles	Frequency in Population at Peak
*rpoS*	RNA polymerase, sigma S (sigma 38) factor	11	7	0.99
*malT*	Fused conserved protein: DNA-binding transcriptional activator/maltotriose-ATP-binding protein	10	26	0.99
*mglD/O (galS/O)*	DNA-binding transcriptional repressor	11	7	0.98
*sdhC*	Succinate dehydrogenase, membrane subunit, binds cytochrome b556	5	1	0.95
*malE*	Maltose transporter subunit	6	5	0.88
*mutL*	Methyl-directed mismatch repair protein	9	2	0.67
*tpiA*	Triosephosphate isomerase	6	1	0.62
*yhaM*	Hypothetical protein	5	2	0.58
*pnp*	Polynucleotide phosphorylase/polyadenylase	3	3	0.39
*asmA*	Putative assembly protein	7	9	0.26
*malS*	Alpha-amylase	4	2	0.16
*hfq*	HF-I, host factor for RNA phage Q beta replication	5	1	0.15
*rcsD*	Phosphotransfer intermediate protein in two-component regulatory system with RcsBC	4	1	0.14
*b3598/mtlA*	Predicted inner membrane protein	5	1	0.09
*murP*	Fused predicted PTS enzymes: IIB component/IIC component	3	5	0.08
*recJ*	ssDNA exonuclease, 5′ → 3′-specific	6	6	0.08
*cusA*	Copper/silver efflux system, membrane component	3	4	0.07
*rhsD*	rhsD element protein	6	14	0.07
*ebgA*	Cryptic beta-d-galactosidase, alpha subunit	6	5	0.06
*eco*	Ecotin, a serine protease inhibitor	3	1	0.06
*amiD*	Putative amidase and lipoprotein	3	2	0.05
*mraY*	Phospho-*N*-acetylmuramoyl-pentapeptide transferase	3	3	0.05
*mrcA*	Fused penicillin-binding protein 1 a: murein transglycosylase/murein transpeptidase	4	5	0.05
*glnG*	Fused DNA-binding response regulator in two-component regulatory system with GlnL: response regulator/sigma54 interaction protein	3	1	0.05
*ytfN*	Hypothetical protein	5	7	0.05
*yfhA*	Putative DNA-binding response regulator in two-component system	6	2	0.05
*fdnG*	Formate dehydrogenase-N, alpha subunit, nitrate-inducible	3	4	0.04
*gltB*	Glutamate synthase, large subunit	5	9	0.04
*sgbE*	l-ribulose-5-phosphate 4-epimerase	4	5	0.04
*metH*	Homocysteine-N5-methyltetrahydrofolate transmethylase, B12-dependent	4	8	0.04
*polB*	DNA polymerase II	4	3	0.04
*cls*	Cardiolipin synthase 1	3	4	0.04
*ybhF*	Fused predicted transporter subunits of ABC superfamily: ATP-binding components	3	2	0.04
*purL*	Phosphoribosylformyl-glycineamide synthetase	5	8	0.04
*thrA*	Fused aspartokinase I and homoserine dehydrogenase I	4	6	0.04
*frdA*	Fumarate reductase (anaerobic) catalytic and NAD/flavoprotein subunit	3	1	0.04
*recG*	ATP-dependent DNA helicase	6	8	0.04
*trxB*	Thioredoxin reductase, FAD/NAD(P)-binding	3	4	0.04
*malZ*	Maltodextrin glucosidase	4	4	0.03
*mdtC*	Multidrug efflux system, subunit C	3	5	0.03
*flu*	CP4-44 prophage; antigen 43 (Ag43) phase-variable biofilm formation autotransporter	3	4	0.03
*treB*	Fused trehalose(maltose)-specific PTS enzyme: IIB component/IIC component	3	2	0.03
*entF*	Enterobactin synthase multienzyme complex component, ATP-dependent	3	5	0.03
*fadD*	Acyl-CoA synthetase (long-chain-fatty-acid–CoA ligase)	3	4	0.03
*entS*	Putative transporter	3	4	0.03
*phnF*	Putative DNA-binding transcriptional regulator of phosphonate uptake and biodegradation	3	3	0.03
*pqiA*	Paraquat-inducible membrane protein A	3	4	0.03
*kdpB*	Potassium translocating ATPase, subunit B	3	4	0.03
*puuP*	Putrescine importer	3	2	0.03
*dnaX*	DNA polymerase III/DNA elongation factor III, tau and gamma subunits	4	4	0.03
*lhr*	Putative ATP-dependent helicase	3	4	0.03
*astD*	Succinylglutamic semialdehyde dehydrogenase	3	3	0.03
*gabP*	Gamma-aminobutyrate transporter	3	3	0.03
*ytfF*	Putative inner membrane protein	3	1	0.03
*phnK*	Carbon-phosphorus lyase complex subunit	4	4	0.03
*gutQ*	Putative phosphosugar-binding protein	3	2	0.03
*acs*	Acetyl-CoA synthetase	5	4	0.03
*narZ*	Nitrate reductase 2 (NRZ), alpha subunit	3	4	0.03
*chaA*	Calcium/sodium:proton antiporter	3	1	0.03
*ligB*	DNA ligase, NAD(+)-dependent	3	5	0.03
*nfrA*	Bacteriophage N4 receptor, outer membrane subunit	3	5	0.03
*fdoG*	Formate dehydrogenase-O, large subunit	3	3	0.03
*carB*	Carbamoyl-phosphate synthase large subunit	4	4	0.02
*yfbS*	Putative transporter	4	3	0.02
*yhgF*	Putative transcriptional accessory protein	3	4	0.02
*rseP*	Zinc metallopeptidase	3	3	0.02
*ybaP*	Hypothetical protein	3	2	0.02
*lpxM*	Myristoyl-acyl carrier protein (ACP)-dependent acyltransferase	5	1	0.02
*ulaG*	Putative l-ascorbate 6-phosphate lactonase	3	3	0.02
*dam*	DNA adenine methylase	3	2	0.02
*yhiN*	Putative oxidoreductase with FAD/NAD(P)-binding domain	3	2	0.02
*garK*	Glycerate kinase I	3	4	0.02
*acnB*	Bifunctional aconitate hydratase 2/2-methylisocitrate dehydratase	3	2	0.02
*trpD*	Fused glutamine amidotransferase (component II) of anthranilate synthase/anthranilate phosphoribosyl transferase	4	3	0.02
*uup*	Fused predicted transporter subunits of ABC superfamily: ATP-binding components	3	3	0.02
*hisB*	Fused histidinol-phosphatase/imidazoleglycerol-phosphate dehydratase	3	2	0.02
*yphD*	Putative sugar transporter subunit: membrane component of ABC superfamily	3	3	0.02
*tauB*	Taurine transporter subunit	3	3	0.02
*creC*	Sensory histidine kinase in two-component regulatory system with CreB or PhoB, regulator of the CreBC regulon	4	2	0.02
*yidR*	Hypothetical protein	3	3	0.02
*ydcR*	Fused predicted DNA-binding transcriptional regulator/predicted amino transferase	3	2	0.02
*cpsB*	Mannose-1-phosphate guanyltransferase	3	2	0.02
*eutB*	Ethanolamine ammonia-lyase, large subunit, heavy chain	3	3	0.02
*proA*	Gamma-glutamylphosphate reductase	3	3	0.02
*sseB*	Rhodanase-like enzyme, sulfur transfer from thiosulfate	3	3	0.02
*rutF*	Putative oxidoreductase, flavin:NADH component	3	3	0.02
*aer*	Fused signal transducer for aerotaxis sensory component/methyl accepting chemotaxis component	3	2	0.02
*mglC*	Methyl-galactoside transporter subunit	3	2	0.02
*ygjK*	Putative glycosyl hydrolase	3	2	0.02
*ybhG*	Putative membrane fusion protein (MFP) component of efflux pump, membrane anchor	3	2	0.02
*hisD*	Bifunctional histidinal dehydrogenase/histidinol dehydrogenase	3	1	0.02

^a^The entries include single nucleotide substitutions and 1-base insertions/deletions in coding sequences.

### Dynamics of Phenotypic and Genotypic Adaptation

**Fig. 2.—:**
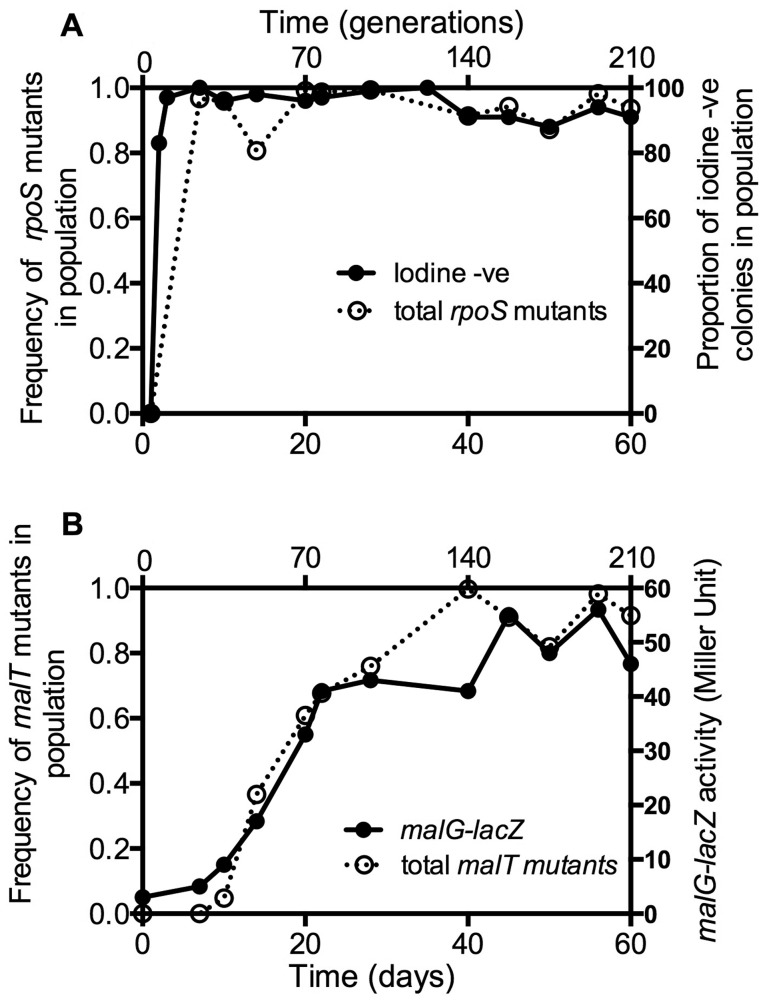
Correlation between phenotypic and genotypic sweeps. (*A*) The frequency of isolates from the population showing altered phenotype (iodine staining negative, •) and the sum of all the different *rpoS* mutations (○) in the population at given time points during 60 days of glucose-limited continuous culture as shown in supplementary table S1, Supplementary Material online. (*B*) Changes in expression of the *mal* regulon (*malG–lacZ* activity of culture, •) and the sum of the different *malT* mutations (○) in the population at given time points. The proportion of the population with iodine-negative phenotype and the *malG–lacZ* activity of the culture were estimated as described in the Materials and Methods section.

We undertook phenotypic screening of the whole population for commonly acquired adaptations in our chemostat system. Phenotypes caused by mutations in *rpoS* (altered glycogen staining) and *malT* (altered *mal* gene expression) showed that the sweeps affecting *rpoS* and *mal* gene expression started before 7 and after 14 days, respectively (fig. 2). These results are consistent with previous chemostat studies initiated with the same ancestral strain using identical culture conditions (Notley-McRobb et al. 2003), thus this single population was typical of other previous shorter-run chemostat cultures.

In order to check whether the phenotypic sweeps match the genotypic sweeps, we plotted the total frequency of all *rpoS* and *malT* nonsynonymous alleles in the population. There are 7 and 26 alleles of *rpoS* and *malT,* respectively, in the population (table 1). As shown in figure 2, the sum contribution of all the *rpoS* and *malT* alleles combined matches the phenotypic dynamics due to mutations in these genes. So the dynamics of mutational change in the metagenome correlates well with the independent phenotypic sweeps.

**Fig. 3.—:**
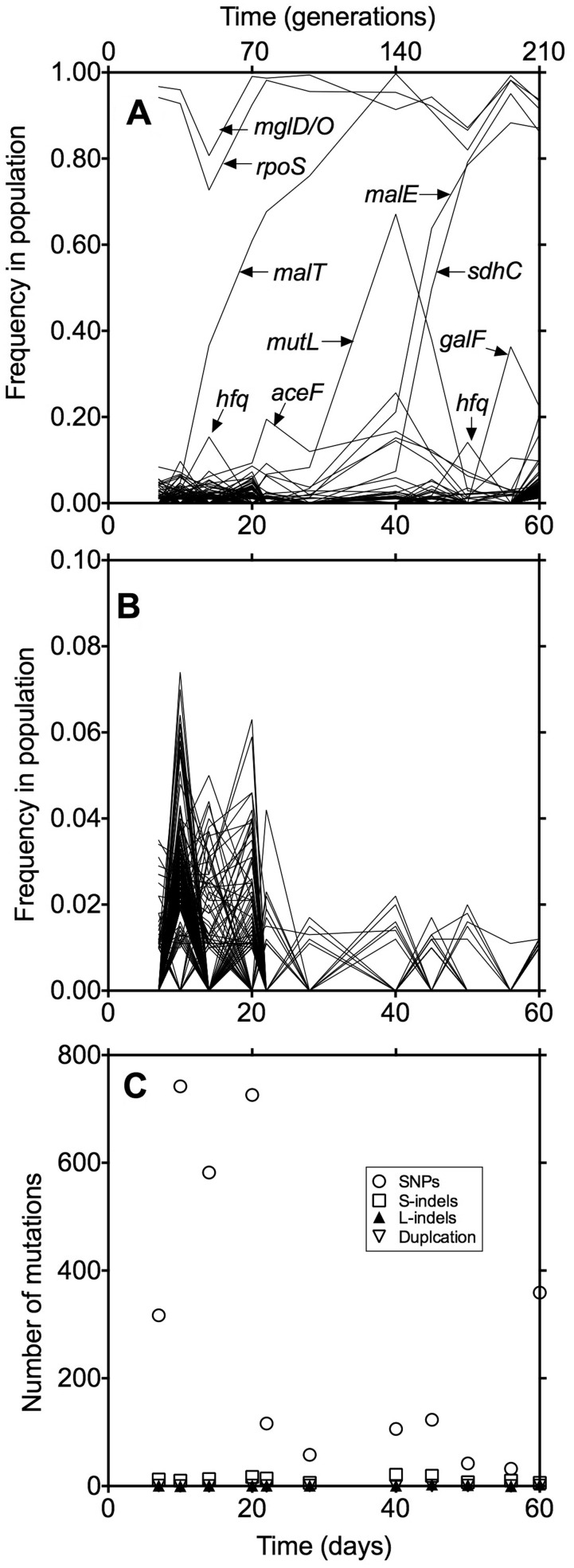
Dynamics of mutational changes in a chemostat population. (*A*) The total collection of the 2,709 mutations in 1,717 genes or regions identified in a glucose-limited chemostat population of *E. coli* K-12 strain BW2952. Each line represents a gene in which a mutation had occurred. (*B*) An extended view of the subset of genes from (*A*) in which mutant sequences were present in less than 20% of the population during the 60 days. (*C*) The total load of mutations in the population over 60 days. The counts of SNPs, single base-pair indels (S-indels), large indels (L-indels), and duplication mutations that were detected in at least 1% of the total population across 11 different samples collected over 60 days of continuous culture are shown. Frequencies of each mutation were estimated from the number of reads and total coverage at that particular genome position.

To gain further insight into genotype dynamics, we plotted the frequency of mutations in all 1,717 genes containing sequence changes (fig. 3). In figure 3*B*, for clarity we show a subset of the data in figure 3*A* at a 10-fold magnified scale and show the vast number of mutations that occur transiently, particularly before day 20. The mutation numbers in each sample are summed in figure 3*C*, which also shows that the total number of mutations decreases after 20 days, but at least 42 different mutations remain throughout the course of the 60 days.

### Individual Gene Dynamics and Allele Frequencies

As shown in figure 3*A*, it is evident that already at day 7, a high proportion of the population had acquired mutations in *rpoS* and *mgl* genes; such strongly beneficial mutations have appeared reproducibly in all glucose-limited populations of BW2952 in 3–5 days at this dilution rate (Notley-McRobb et al. 2003; Ferenci 2008b) and are also consistent with the phenotypic sweep shown in figure 2. Over the remaining 54 days, the metagenome continued to include mutations in *rpoS* and *mgl* in over 80% of bacterial sequences, although never to 100% fixation. The only time-points at which the *rpoS* and *mgl* mutations drop below 80% are at days 14 and 50, when *hfq* mutations transiently increase to greater than 10% abundance. As shown before (Maharjan and Ferenci 2013), *hfq* mutants remain *rpoS*^+^*mgl*^+^ explaining the reciprocal blips in the *rpoS*/*hfq* proportions (fig. 3*A*).

**Fig. 4.—:**
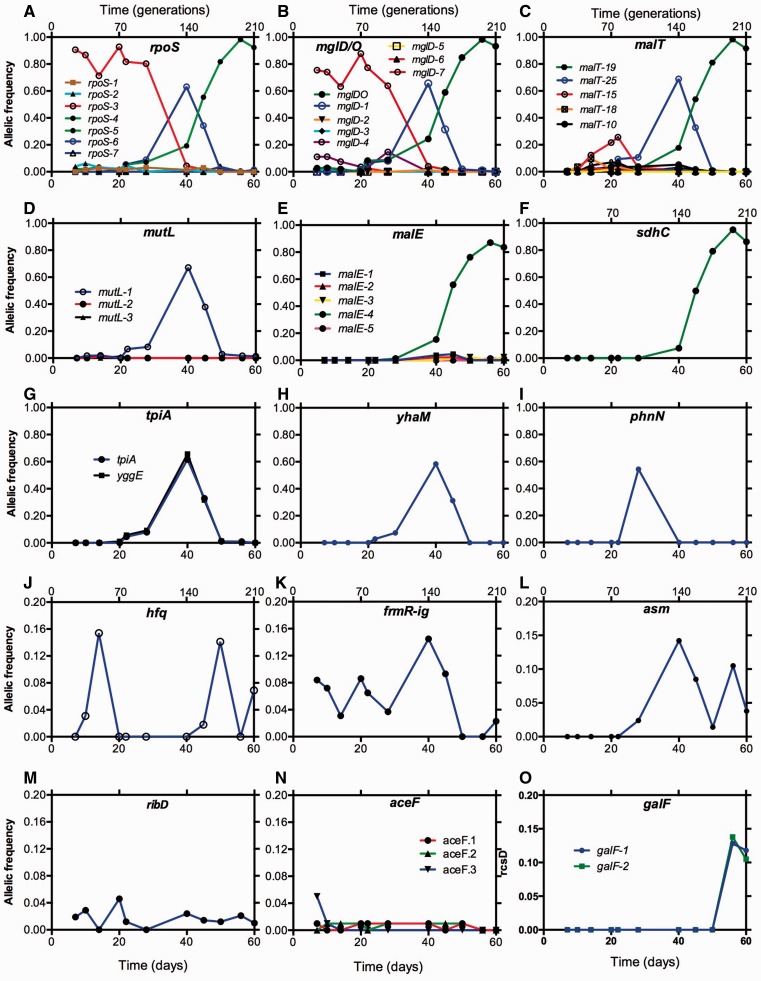
Dynamics of frequency changes of alleles within individual genes. The genomic position of each mutation in each gene is listed in the supplementary table S1, Supplementary Material online. Labels are shown for only the more dominant 5 of the 26 nonsynonymous *malT* alleles.

Another significant sweep was the *malT* change from day 14, which also, as previously observed (Notley-McRobb and Ferenci 1999b), became highly represented for the rest of the experiment. The *malT* sweeps were then followed by *malE* and *sdhC* mutations (figs. 3 and 4); the possible role of these mutations is discussed below. These were not previously seen in chemostats but occurred after 40 days, beyond the 28 days sampled in earlier studies.

The most unique change occurred concurrently in several genes, peaking at days 40–45 in greater than 50% of the population and included a *mutL* mutation. Mutations in this gene result in a mutator phenotype and so it is likely that the other exactly coincident peaks (in *yggE, tpiA*) were linked with the mutator genome when it increased in frequency. In turn, as shown below in figure 4*E*, the enrichment of the mutator-associated mutations is most likely due to the benefit that other mutations such as *rpoS*, *mglD,* and *malT* provided at day 40 but this lineage was outcompeted by other members of the same population with different beneficial combinations. Independent whole-genome sequencing of a clone isolated from the day 40 sample showed that the *mutL*-containing genome also has mutations in *rpoS*, *mglD, malT, yggE* and *tpiA,* which further confirms their linkage*.* Mutator phenotypic assay showed that this clone also had an elevated rate (>100-fold) of spontaneous mutation. Fitness and other phenotypic contributions of *yggE* and *tpiA* encoding YggE hypothetical protein and triosephosphate isomerase, respectively, are unknown. This *mutL* finding increases to three the mutator types found in chemostat populations (*mutS* and *mutY* were previously found [Notley-McRobb, Seeto, et al. 2002]) so this form of temporary enrichment of mutators is a common feature of a rapidly evolving population.

Another mutation appeared in greater than 50% of the population, in *phnN* (fig. 3*B*). This transient peak in *phnN* at day 28 exactly coincided with the appearance and disappearance of a *malT* mutation at position 3440466 (*malT-18*) (see fig. 3*C* and *E*) so probably was genetically hitchhiking with this equally transient allele. It is not obvious why *phnN* would have a role under glucose limitation, being involved in phosphonate utilization that is cryptic in *E. coli* K-12 (Hove-Jensen et al. 2003).

### Smooth Phenotypic Sweeps Are Genetically Complex

In figure 4*A*–*C*, we can observe how multiple soft sweeps involving *rpoS*, *mgl,* and *malT* mutations occur in unprecedented detail. Most interestingly, the net sweeps reducing the ancestral proportion of wild-type sequences shown in figures 2 and 3 are caused by a succession of different combinations of the mutations in *rpoS*, *mgl,* and *malT*. There were 7–26 different alleles of the genes that appear and disappear together over the 60 days even though the total population load of *rpoSmglmalT* triple mutants exceeded 80% over extended periods (fig. 3). The presence of multiple alleles of these genes is strongly indicative of soft sweeps in the population. In the large population studied, many independent strongly beneficial mutations can arise and changes at several sites within a gene can satisfy the fitness gain; in *malT*, *mglD* and *rpoS*, this is certainly the case (Notley-McRobb and Ferenci 1999a, 1999b; Notley-McRobb, King, et al. 2002).

The first dominant *rpoS-3* allele was a mutation at genome position 2751003, prominent already at day 7. Minor proportions of alleles such as *rpoS-2* were also present early on. Other minor alleles of *rpoS* such as *rpoS-4* were barely detectable prior to day 20 but became the dominant allele by day 56 (fig. 4*A*). In between, the first dominant allele, *rpoS-3,* was eliminated by successive sweeps of *rpoS-4* and then *rpoS-6*, which had a sharp, transient peak at day 40. The loss of function mutations in *rpoS* at each position does not have a significant fitness difference, as evidenced by iodine staining, so the rapid alterations in the proportion of dominant *rpoS* alleles must have been due to additional mutations; the combinations leading to the sweeps can be deduced from the concurrent frequencies of other mutations as shown below.

**Fig. 5.—:**
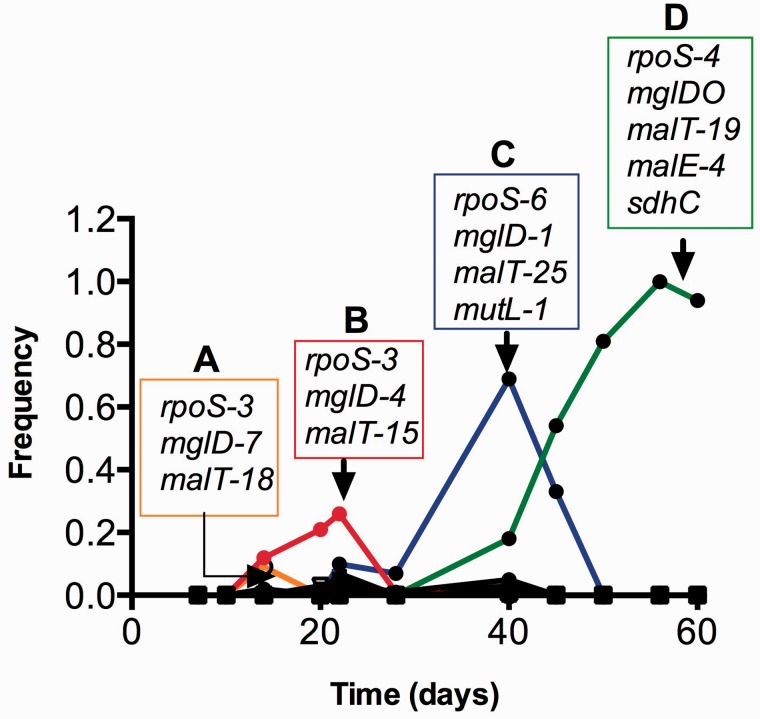
Summary of events superimposed on the trajectories of *malT* allele frequencies. The mutations peaking at particular times are here superimposed on the *malT* allele sweeps shown in figure 4*C* (*malT* panel) to show the combinations of mutations participating in replacements over the 60 days. The association of different alleles of *rpoS, mglD, malT, malE,* and *sdhC* were based on the allelic frequency dynamics shown in figure 4 and verified by sequencing of individual isolates (see text for more detail).

The *mgl* profile in figure 4*B* showed a near-identical pattern of allele successions to that of *rpoS*. The first dominant *mgl* mutation was at position 2132151 (*mglD-7*), already by day 7. Another *mgl* allele (*mglD-4*) present in lower proportions at early times was at position 2132020 (fig. 4*B*). The proportions of these alleles in combination match that of *rpoS-3*, suggesting that the strain with *rpoS-3* diverged into *rpoS-3mglD-7* (dominant) and *rpoS-3mglD-4* (minor) before day 7. From day 28, another allele combination, *rpoS-6mglD-1* swept to over 60% abundance, reducing the proportion of the earlier *rpoSmglD* combinations. Further, the final dominant *mgl* allele (*mglDO*) emerges beyond day 28, with an *mgl*O operator mutation in combination with yet another *rpoS* allele, *rpoS-4*. The proportion of the individual *rpoS* and *mgl* mutations contributing to the paired alleles was near identical and their trajectories were also remarkably similar, indicating the linkage of the alleles in the same genome. These sweeps are indicated in a summary form shown in fig. 5.

The benefit of these *rpoS* and *mgl* combinations still does not explain the frequency changes, until one considers the additional sweeps of *malT* and other mutations in these backgrounds as summarized in figures 4 and 5. Mutations in *malT* became evident after day 10. Mutations at positions 3440466 (*malT-18*) and 3440328 (*malT-15*) reached 10–20% abundances together the coincident *rpoS-3mglD-7* (group A in fig. 5) and *rpoS-3mglD4* (group B in fig. 5). At day 40, the transient peak with the *rpoS-6mglD-1* combination coincided exactly with the spread of the *malT-25* allele, both in scale and in profile (fig. 4*C*). Thus, it is most likely that the *rpoS*-6*mglD-1 malT-25* (group C in fig. 5) combination was central to the sweep between days 28 and 40. As mentioned earlier, also coincident at day 40 was *mutL*. Thus, *mutL* and other associated genes must have hitchhiked together with *rpoS*-6*mglD-1 malT-25* allelic combination.

The combination of mutations dominant at day 60 had yet another set of allele combinations in *rpoS*, *mgl,* and *malT*. The major sweep between days 40 and 60 contained the *rpoS-4*, *mglO* and *malT-19* alleles, with *malT-19* becoming prominent only after day 28 (fig. 5). Also spreading simultaneously with the same trajectory were *malE* with mutation at 4183156 (*malE-4*) and *sdhC* mutation at 657360. This *rpoS-4mglOmalT-19malE-4sdhC* (group D in fig. 5) combination resulted in the drastic reduction of all other previously appearing *rpoS*, *mgl,* and *malT* alleles in this population. The combination of the five mutations in one isolated clone from day 60 shown in figure 5 was confirmed by PCR of each gene and Sanger sequencing. The events at days 56–60 come closest to a purifying sweep in this experiment but even here the population still contained a significant proportion of *hfq* and other mutational lineages (fig. 4*J*–*O*), maintaining persisting diversity in the culture. The numerous low-frequency mutations (fig. 3*B*) also provided a rich source material for further evolution.

### The Persistence of Low-Abundance Alleles of the Same Gene

As shown in figure 4*J–O*, multiple genes besides the above-mentioned *hfq* were altered at multiple time-points and in significant numbers. Several of the mutations shown in figure 4 were consistently present at or above the 1% abundance, without being eliminated by other fluctuations in the population. The persistence under glucose limitation suggests a fitness benefit of these mutations without a capacity to sweep through the whole population; this is best explained by a frequency-dependent fitness as was demonstrated for *hfq* (Maharjan, McKenzie, et al. 2013). Curiously, three alleles of *aceF* and two of *galF* were simultaneously present in the population. Only the last major change in frequency of other genes toward day 60 reduced the numbers of *aceF* mutants in the population, but the others shown in figure 4 were maintained and indeed rose in the case of *galF*.

*AceF* is a subunit of pyruvate dehydrogenase so mutations in *aceF* could change central metabolism; *aceF* mutants can for example have elevated pyruvate levels in cells (Tomar et al. 2003). It is not clear how this could change fitness under glucose limitation but is the kind of change that could give rise to cross-feeding consortia as seen in long-running chemostats (Rosenzweig and Adams 1994). As shown in figure 4*B*, some other mutations in *asm, galF, ribD,* and near *frmR* were persistently present over many samples until day 60, but never exceed 20% of the population. The apparently frequency-dependent benefit of these mutations is so far unidentified.

### The Persistence of Low-Abundance Indels in the Chemostat Population

**Fig. 6.—:**
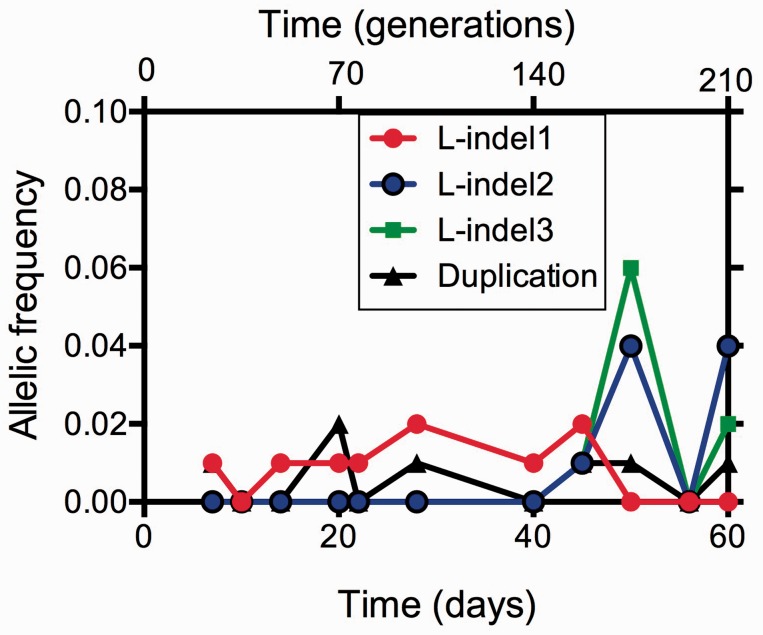
Dynamics of frequency changes of each large indel and duplication mutation found in the chemostat population. The genomic position of each mutation in each gene is listed in the supplementary table S1, Supplementary Material online.

As shown in figure 6, four larger genome changes, including four large insertions and a tandem duplication, also persisted as minority types over multiple samples. Of the five large mutations (4 L-indels and 1 duplication), four persisted as minority types over multiple time points (fig. 6 and supplementary table S1, Supplementary Material online). One of the four large insertions (L-indel1) was located inside *mglD,* so it is likely to have the same beneficial role as the other *mgl* mutations found as SNPs (fig. 4*B*) causing loss of MglD repressor function. L-indel 2 and L-indel 4 were located in *hyfA* and *ydeK,* respectively. These encode hydrogenase 4, 4Fe–4S subunit and a putative lipoprotein, whereas L-indel 3 was located in an intergetic region near *dnaK*, a molecular chaperone protein. The large tandem duplication comprised 23,060 bp (positions 1301981–1325042 of the ancestral BW2952 genome), which contains 25 genes. The role of the duplication of these genes under glucose-limitation is unknown and covers no obviously beneficial functions.

## Discussion

We demonstrated that apparently simple phenotypic sweeps can hide complex genetic changes in populations. As most clearly seen with *rpoS* over 60 days, the constantly high abundance of the low-RpoS phenotype was due to the successive contribution of three distinct *rpoS* mutations in combination with other beneficial mutations. Each of these alleles arose directly from the ancestor, but became more dominant when additional mutations were acquired. The richness of the population in *rpoS* mutations extends beyond the three prominent alleles and at least five other alleles could be detected at lower frequencies; other alleles were probably there but below the detection limit of metagenomics. In all the alleles in the population, there was phenotypic convergence as to the benefit of *rpoS* mutations, but the combination of different alleles with different other mutations contributes to the complexity of fitness pathways in the evolving population. Likewise, 8 *mgl* mutations and 26 *malT* mutations contribute to the deceptively smooth phenotypic behavior (fig. 2) but highly lumpy mutational landscape in the evolving population (fig. 5). Simple assumptions about 1:1 relationships between phenotypic sweeps and mutations are therefore unwarranted, especially in large populations.

Our results are consistent with the contribution of multiple alleles and soft sweeps to most, but not all, adaptations in a large bacterial population. The significant frequency changes resembling hard sweeps and originating from single alleles were associated with the *mutL* and *sdhC* mutations (fig. 4*D* and *F*, respectively). *sdhC* is the only single-allele gene rising from 1% to greater than 90% frequency in the population in a single sweep. The *mutL* case is more misleading however, because mutator mutations are not themselves beneficial and thus hitchhiked with other beneficial mutation(s) at day 40. It is remarkable that a mutator mutation, thought to be important when evolution is limited by mutation supply (de Visser et al. 1999) actually becomes common even in a large population where every single nucleotide mutation will occur nearly ten times every generation.

One of the most fascinating aspects of the multiple alleles involving *malT*, *mgl,* or *rpoS* was that each mutation appeared from an undetectably low base frequency even late in the population and not from other, more frequent types. This is consistent with the notion that there is a significant pool of bacteria with mutations at a low frequency in the population from which most of these sweeps emerged. In the culture studied, it is worth recalling that 1% frequency is still a subpopulation of 10^8^ cells, large enough to be a source of further mutational innovations. Our results do not resemble any of the proposed models recently discussed (e.g., fig. 2 in Barrick and Lenski [2013] or fig. 1 in Herron and Doebeli [2013]), all of which assume that beneficial mutations repeatedly occur consecutively in the majority types in a population. In our study, all the successful later lineages emerged from the heterogeneity in the population present at less than 1% abundance in the population.

Periodic selection, as seen in the fluctuation in the proportion of neutral mutations in a population, is a long-observed feature of evolving bacterial populations (Atwood et al. 1951b). Because periodic selection events are operationally described by the fluctuation in the proportion of a neutral marker (such as T5 resistance) in the population, they describe population changes due to all mutational sweeps, even those with mutations in the same gene. The fluctuations in neutral mutations in chemostats have been described in detail (Atwood et al. 1951a; Kubitschek 1970). A characteristic of these periodic events is that they are frequent and only partial in reducing the proportion of neutral mutations. As an example, our own published experiments in following periodic selection events (Notley-McRobb and Ferenci 2000; Notley-McRobb et al. 2003) demonstrated, on reanalysis of the data, that there was approximately 1 sweep/generation at 0.1 h^−^^1^ dilution rate as used in this study. We can now see how the multiple frequency changes involving many genes (figs. 3 and 4) determine frequent periodic selection events and changes in genotype and phenotype in a population. Multiple changes in 23 genes with population frequency shifts exceeding 10% were evident from figures 3 and 4 and multiple combinations of mutations contributed to the number of fluctuations in this large population. Our results are thus consistent with classic observations that periodic selection events are numerous, show only partial sweeps and are indeed more frequent than can be explained by 1-gene:1-mutation combinations. The observation that different combinations of mutations contributed to population shifts means that it is unclear whether true clonal interference (de Visser and Rozen 2006; Barroso-Batista et al. 2014) is operating in this population. Our results do however strongly resemble recent results with yeast populations in that multiple mutations arise and move synchronously through the population as mutational “cohorts” (Lang et al. 2013).

This high-coverage metagenomics analysis identified a large number of mutations spreading in glucose-limited populations. One of the most common new mutations, in *malE*, may provide a benefit by reducing the load imposed by the *malT* mutations widespread in the population. The *malT* constitutivity mutation results in high levels of not only LamB protein (beneficial because this increases the transport of limiting glucose [Death et al. 1993]) but also other *mal* regulon proteins including MalE (which is not of benefit). MalE is a protein made in high amounts and has the cost of having to be secreted into the periplasmic space (Kellermann and Ferenci 1982). A likely benefit of its loss is thus the alleviation of the cost of producing large amounts of an unneeded protein. Another possibility is that loss of MalE alleviates competition in protein secretion, because useful proteins such as LamB need to be exported from the cytoplasm in large amounts through the same secretion pathway (Misra et al. 1991).

We do not know the physiological benefit of several of the other mutations identified here, such as in *acs* and *aceF* (table 1). Given the role of these gene products in central metabolism affecting the tricarboxylic cycle, it can be proposed that metabolic adaptations can provide a benefit under glucose limitation. Indeed, an alternative to the TCA cycle, the glyoxylate cycle, has been implicated in glucose-limited growth so a speculative explanation is a beneficial switch between these pathways (Fischer and Sauer 2003; Maharjan et al. 2005). The nature of these benefit(s) will need to be investigated by detecting changes in metabolic fluxes in the mutant strains. There are also less obvious benefits under glucose limitation among the mutations present in table 1 in multiple samples, such as in *galF* and the putative efflux protein *mdtG* as well as the indels in figure 6. Further physiological analysis is needed to identify these benefits.

The single most successful combination of mutations peaked at day 56 and analysis of individual isolates purified from the population confirmed the combination of mutations in the dominant type shown in figure 5. At that time, the population came closest to a purifying sweep with the major type close to 100% abundance; nevertheless the minority types carrying *hfq*, *asm,* and *ribD* mutations were largely unaffected at days 56–60. Two large insertions and a tandem duplication also persisted through day 60 (fig. 6). From the metagenomics, we do not know whether any or all of these low-abundance mutations occurred in combination in the same cell to provide the fitness benefit under glucose limitation.

Crucially from the point of view of maintained diversity, the total number of mutations in the population did not continue to decrease (fig. 1) and the continued presence of greater than 40 mutations in the population over time indicates that a diverse range of mutations is maintained in the population. The mutation supply and the minority lineages in figures 4 and 5 provide the genetic potential for further evolution as well as the maintenance of heterogeneity during the later phases of this experiment.

In conclusion, high coverage metagenomic analysis has revealed in unprecedented detail the multiple soft sweeps in an evolving *E. coli* population and the way these contribute to the sum total of phenotypic changes. The results explain the early findings on periodic selection events and why incomplete sweeps were previously found. The data have identified several low-proportion mutations not previously found in evolving chemostat populations and have established that minority alleles can persist stably for hundreds of generations in independent lineages.

## Supplementary Material

Supplementary table S1 is available at *Genome Biology and Evolution* online (http://www.gbe.oxfordjournals.org/).

Supplementary Data
