# Supplementary material for: Simple Phenotypic Sweeps Hide Complex Genetic Changes in Populations
Source: Genome Biol Evol. 2015 Jan 13;7(2):531–44. doi: 10.1093/gbe/evv004 (PMC4350175; doi:10.1093/gbe/evv004)
Supplement: Supplementary Data [file supp_7_2_531__index.html]

Simple phenotypic sweeps hide complex genetic changes in populations — Simple Phenotypic Sweeps Hide Complex Genetic Changes in Populations — Supplementary Data 

# Simple Phenotypic Sweeps Hide Complex Genetic Changes in Populations

## Supplementary Data

files

**Files in this Data Supplement:**

- Supplementary Data - xlsx file
